# The Acute Inflammatory Response to Absorbed Collagen Sponge Is Not Enhanced by BMP-2

**DOI:** 10.3390/ijms18030498

**Published:** 2017-02-25

**Authors:** Hairong Huang, Daniel Wismeijer, Ernst B. Hunziker, Gang Wu

**Affiliations:** 1 Department of Oral Implantology and Prosthetic Dentistry, Academic Centre for Dentistry Amsterdam (ACTA), University of Amsterdam and Vrije Universiteit Amsterdam, Amsterdam Movement Sciences, Gustav Mahlerlaan 3004, 1081LA Amsterdam, The Netherlands; hhrstudy@126.com (H.H.); d.wismeijer@acta.nl (D.W.); 2 Departments of Osteoporosis and Orthopaedic Surgery, Inselspital (DKF), University of Bern, Murtenstrasse 35, 3008 Bern, Switzerland; ernst.hunziker@dkf.unibe.ch

**Keywords:** bone morphogenetic protein-2 (BMP-2), absorbed collagen sponge (ACS), inflammation, vascularization, biomechanical

## Abstract

Absorbed collagen sponge (ACS)/bone morphogenetic protein-2 (BMP-2) are widely used in clinical practise for bone regeneration. However, the application of this product was found to be associated with a significant pro-inflammatory response, particularly in the early phase after implantation. This study aimed to clarify if the pro-inflammatory activities, associated with BMP-2 added to ACS, were related to the physical state of the carrier itself, i.e., a wet or a highly dehydrated state of the ACS, to the local degree of vascularisation and/or to local biomechanical factors. ACS (0.8 cm diameter)/BMP-2 were implanted subcutaneously in the back of 12 eight-week-old Sprague Dawley rats. Two days after surgery, the implanted materials were retrieved and analysed histologically and histomorphometrically. The acute inflammatory response following implantation of ACS was dependent of neither the presence or absence of BMP-2 nor the degree of vascularization in the surrounding tissue nor the hydration state (wet versus dry) of the ACS material at the time of implantation. Differential micro biomechanical factors operating at the implantation site appeared to have an influence on the thickness of inflammation. We conclude that the degree of the early inflammatory response of the ACS/BMP-2 may be associated with the physical and chemical properties of the carrier material itself.

## 1. Introduction

Recombinant human bone morphogenetic protein-2 (BMP-2), a member of the transforming growth factor beta (TGF-β) superfamily, is in clinical use since more than a decade [1,2]. It is used in clinical practice for spinal fusion [3] and for treatment of non-unions to enhance bone formation processes and to accelerate the bony healing response; in dental practice it is used for oral and maxillofacial reconstruction [4,5].

Even though the clinical use of BMP-2 is very successful, its clinical application is associated with some serious unwanted effects such as heterotopic bone formation [6], bone resorption (by osteoclast activation) and formation of cyst-like bone voids [7], as well as postoperative inflammatory swelling [8,9] and neurological symptoms, etc.

BMP-2 is clinically applied as a free factor (Infuse^®^ (USA), Inductos^®^ (Europe)) together with an ACS as a carrier. BMP-2 of this product is used in very high dosage, and it is believed that it is this high dosage level of BMP-2 that leads to extensive inflammatory responses. This use-associated inflammation is one of the main reasons why several of the above-described unwanted effects do occur. It is also believed by many authors that BMP-2 itself contributes significantly to the enhancing of the inflammatory response during and after the implantation of the construct in this kind of a tissue engineering approach. Indeed, several publications report that BMP-2 itself enhances the swelling and the inflammatory response in the surrounding soft tissues in conjunction with the carrier material (ACS) [10].

Seroma formation is, for example, a frequently observed side effect of BMP-2-use, encountered most commonly in the first week postoperatively, as described in several studies [8,11]. Rihnet et al. [12] found that lumbar seromas occurred in 1.2% of rhBMP-2 treated patients compared to 0% in the control patient population. Robin et al. [8] described postoperative seroma formations associated with BMP-2 use in the surrounding soft tissues in the cervical region that led to bilateral paresthesia of the upper extremities. In clinical cases with BMP-2-induced seromas, elevated serum levels of inflammatory cytokines were found, such as those of IL-6, IL-8, and TNF-α [13], as well as those of IL-10 [10].

Indeed in the publication of Lee et al. [10], a dose-dependency of the inflammatory response to high dosage levels of BMP-2 was found. However, in a report of Wu et al. [14] it was described that BMP-2, in particular when delivered in a slow release system, is able to attenuate inflammatory responses. In other in vivo animal experiments [15], microcomputed tomography and histological analyses confirmed that PCL/PLGA/collagen/rhBMP-2 scaffolds (long-term delivery mode) showed the best bone healing quality at both weeks 4 and 8 after implantation without inflammatory response. Thus, conflicting data are encountered in the scientific literature respecting the role of BMP-2 and it use-associated inflammation.

The purpose of this study was to investigate if the use of BMP-2, when applied at high concentrations as a free factor together with a carrier material (ACS), is indeed associated with a pro-inflammatory response in the acute phase of the body response, i.e., in the initial two days after implantation of this growth factor with the carrier material. It is, indeed, conceivable that it is not the BMP-2 itself that triggers the intensive inflammatory response, but that the inflammation may be elicited by a number of other factors operating in close topographical vicinity to the deposited collagen carrier. Such candidate factors may be the degree of tissue vascularity, or the local micromechanical conditions of different physiological stress fields, i.e., depend on differences in the local biological environment (differential niche biology). Another role may be played by the physical state in which the collagen carrier itself is deposited, i.e., inserted in a dry state or in a wet state into the living tissue spaces. Since burst release of BMP-2 (in surgical practise poured onto the ACS sponge) does readily occur, among others due to mechanical manipulation of the construct itself during surgical implantation [16], we set up in our experiments a specific control group in which the collagen carrier was kept in a dry state to assess the possible role of such mechanical stress-modulated release profiles of BMP-2 in the inflammatory response.

In order to clarify the possible role of these various candidate factors, the Sprague Dawley (SD) rat was used as the animal model. ACS carrier material was implanted in the subcutaneous space in the back area (lumbar level). By this set up the deposited collagen carrier patch is exposed on one side towards the skin, where the skin muscles of the rat generate a continuous instability situation, i.e., a high biomechanical instability [17]. On the opposite side of the collagen patch, facing the large underlying lumber muscle package, a relatively stable micromechanical environment is present. In addition, the two different biomechanical niches around these implants are also characterized by specific differential densities of blood vessels. The differential blood vessel densities at these two opposite locations (skin side versus lumbar body side) were quantified in this study in order to elucidate their possible proinflammatory contribution.

## 2. Results

**Figure 1:**
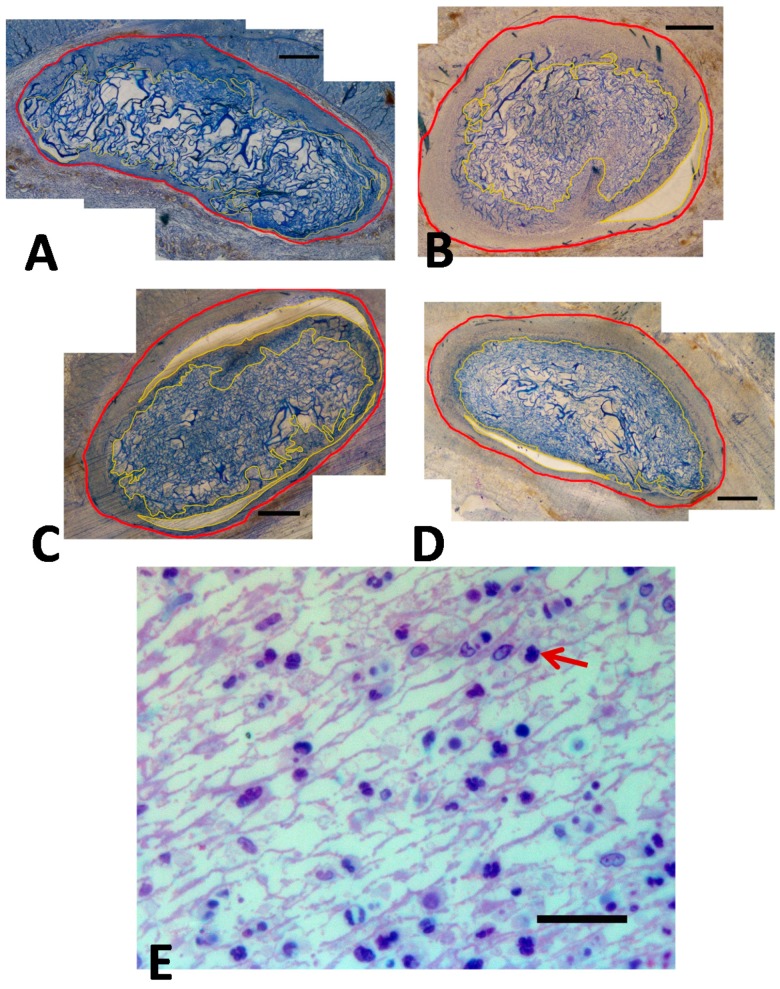
Microscopic findings following subcutaneous implantation of: (**A**) dry Absorbed Collagen Sponge (ACS); (**B**) dry bone morphogenetic protein-2 (BMP-2)/ACS; (**C**) wet ACS; (**D**) wet ACS/BMP-2; (**E**) high magnification of inflamed zone. Red arrow: macrophage. The inflammatory zone was delineated by two different lines: the outer border in red, the inner border in yellow. Bar = 500 µm (in **A**–**D**). The upper side is skin side and the lower side is lumber body side. Numerous macrophages were identified in the highly vascularized inflamed zone (cf. 1E, bar = 20 µm).

Figure 1A–D illustrated that already on the 2nd day after implantation, all collagen implants were surrounded by a capsule of inflamed tissue (delineated by a red line), and was highly vascularized. The inflammatory response involved large numbers of macrophages around each of the implanted collagen sponges (Figure 1E). The outer border of the inflammation border of the collagen implant was delineated by a red line and the inner border of the inflammatory zone by a yellow line (Figure 1A–D).

**Figure 2:**
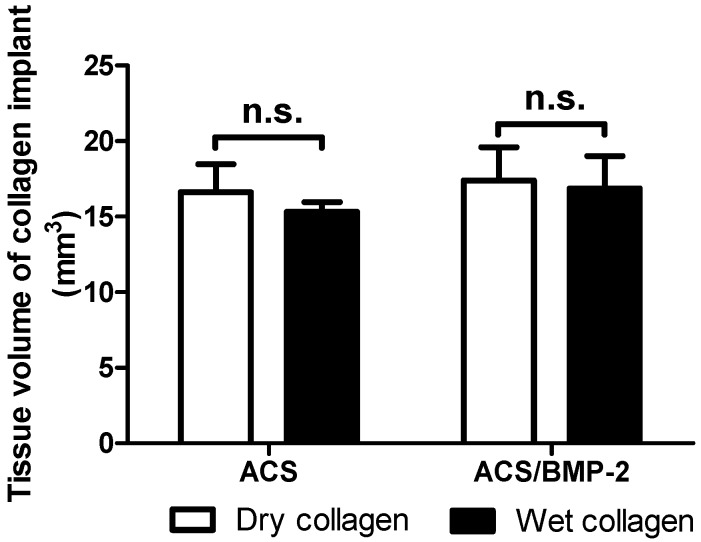
Mean volumes of collagen implants. No significant differences were found between dry ACS and dry ACS/BMP-2 nor between wet ACS and wet ACS/BMP-2. Data were present as Means ± SEM. n.s.: no significant difference.

**Figure 3:**
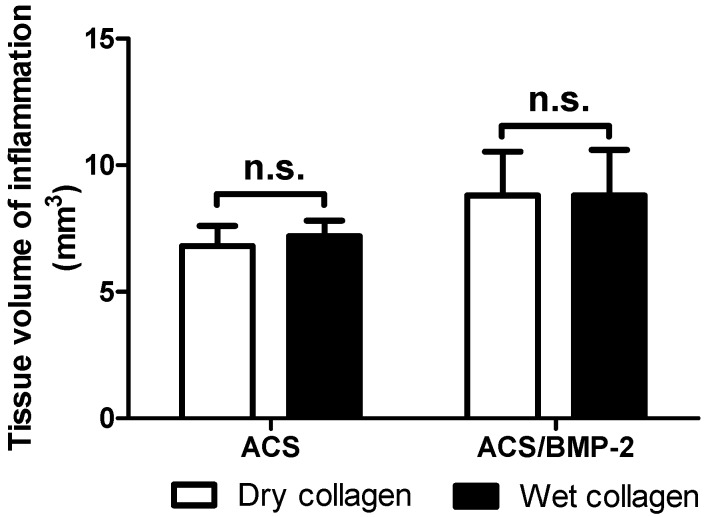
Periimplant inflammation volume. No significant differences were found between dry ACS and dry ACS/BMP-2 nor between wet ACS and wet ACS/BMP-2. Data were present as Means ± SEM. n.s.: no significant difference.

The degree of inflammation activity was gauged by estimation of the volume of the implanted sample and the volume of the inflamed tissue. As Figure 2 and Figure 3 showed, the volumes of the implanted collagen sample and the inflammation area were increased when the carrier (ACS) was loaded with BMP-2. However, there were no significant differences observed between the collagen sponge volumes in the presence or absence of BMP-2, nor if implanted in a wet or a dry (dehydrated) state.

**Figure 4:**
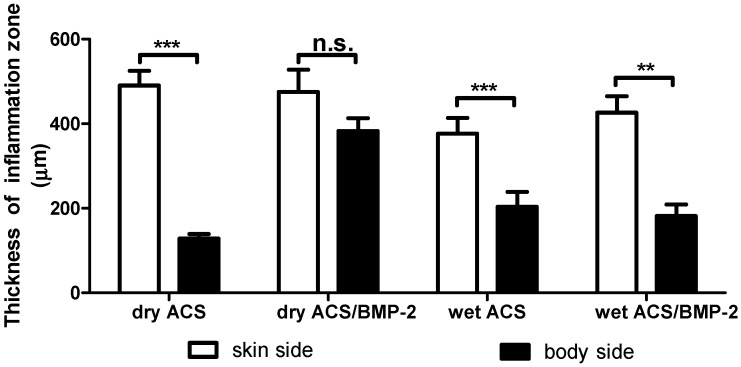
Comparison of the mean thickness of the inflammation zone on the skin side and the body side. There are significant differences in the thickness of the inflammatory zones between the skin side and the lumbar body side in the dry ACS implant group without BMP-2, and in both the wet ACS groups with or without BMP-2. **: *p* < 0.01, ***: *p* < 0.001, n.s.: no significant difference.

As Figure 4 illustrates, the mean thickness of the inflamed tissue at the skin side and the lumbar body side is different, and significant differences were indeed found around the dry ACS implants in the absence of BMP-2 (*p* = 0.001), and in the wet ACS groups in the presence (*p* = 0.0009) or absence (*p* = 0.009) of BMP-2.

**Figure 5:**
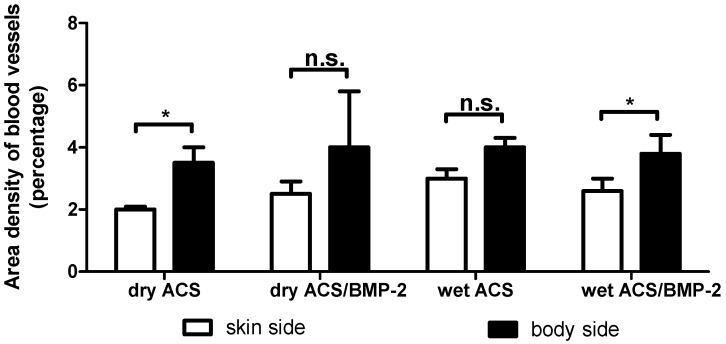
Area density of blood vessels in the dry and wet ACS implant groups, comparing the skin side blood vessel density with the lumbar body side blood vessel density. The data reveal that the density is significantly different physiologically. *: *p* < 0.05, n.s.: no significant difference.

**Figure 6:**
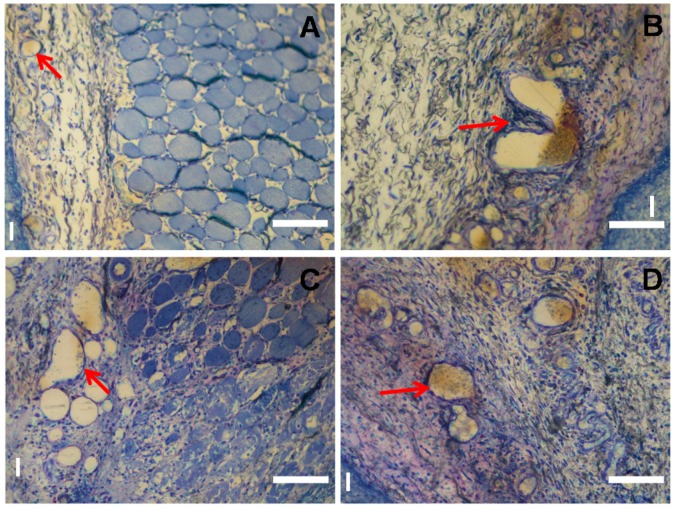
Illustration of blood vessel density in the inflamed area at the skin side (**A**,**C**) and the lumbar body side (**B**,**D**) from the dry (**A**,**B**) and wet (**C**,**D**) ACS. Arrows point to selected blood vessels. I: inflammation area. Bar = 100 µm. Red arrows: blood vessels.

The differential blood vessel densities at these two opposite locations (skin side versus lumbar body side) were quantified in this study in order to elucidate their possible role to contribute to the proinflammatory response. As Figure 5 shows, the area density of blood vessels on both sides were different, the area density of blood vessels in the dry group without BMP-2 on the lumbar body side was significantly higher than that on the skin side (*p* = 0.014) but no significance was found in the wet group. In the group with BMP-2, the area density of blood vessels in the dry group was found to be higher on the lumbar body side than on the skin side, but was not significantly different (due to a high degree of variation; cf. SEM-error bar in Figure 5), in the wet group, the area density of blood vessels on the lumbar body side was significantly higher than that on the skin side (*p* = 0.032). Figure 6 illustrates typical areas and blood vessel densities as encountered on the skin side (Figure 6A,C) and the lumbar body side (Figure 6B,D).

## 3. Discussion

This study is focusing on the initial response of the tissue to the implantation of a sterile scaffold i.e., collagen matrix scaffold, available commercially for use in human patients.

The acute phase of inflammation within the two initial days after implantation is a sterile type of inflammation in the absence of an infection. It is a non-specific tissue response to the foreign body material implanted (carriers, biomaterials) [18]. Moreover, it is associated with tissue swelling, formation of edema as well as the influx of a cell population of the acute inflammatory response type, represented mainly by macrophages, and later on by foreign body giant cells [19]. This inflammatory response is not to be confused with infection, which is caused by foreign agents such as bacteria, viruses, etc. In this study, no infection was observed, and the inflammatory responses were all sterile in nature.

The comparison between wet ACS and dry ACS implanted in the subcutaneously space of rats revealed no difference in extent of inflammation in the acute phase (Figure 3). In addition, the sample size of the ACS, implanted the same way in all experimental groups, exhibited no differences occurring during these two early postimplantation days, i.e., no differences in early degradation activities (Figure 2); also the degree of inflammation, quantified by the inflammation volume around the implanted materials (Figure 3) during this acute inflammation phase did not reveal any significant differences between the control group and ACS/BMP-2 groups. These findings indicate that the acute inflammatory response in such cases is most likely based on the non-specific tissue reactions to foreign materials placed into the body, and it is not dependent on other factors in its extent.

In particular, the comparison between the extent of inflammation in topographically different areas such as the skin area compared to the lumbar body area, which are subjected to different biomechanical stress fields [17], and also to different degrees of vascularity (Figure 5 and Figure 6), that both physiologically do occur at these sites, revealed no differences in the extent of the inflammatory response (Figure 3). This basically implies that the degree of vascularity is irrelevant respecting the extent of the acute inflammation response that can be expected following implantation of foreign materials into the body. The same applies to the state of the hydration of the implant material which is similarly irrelevant to the acute inflammation response with these materials i.e., implanted in a wet hydrated state or implanted into the body in a dry state. Due to the absence of the difference in the inflammatory response in 2 days it is probably implied that the dry material implanted get hydrated very rapidly inside the body so that no difference in inflammatory response can be monitored. However, the thickness of the local inflammation appeared quite irregular in the groups carrying BMP-2, represented by larger coefficient of variation (Figure 5) (dry ACS/BMP-2 group: coefficient of variation (CV) = 100%, coefficient of error (CE) = 45%) The thickness of the local inflammatory response was thus the only factor identified to show any differences between the two chosen topographical locations (skin versus lumbar body), and was thus associated with an asymmetrical response and a high degree of variation (dry ACS/BMP-2 group: CV = 100%, CE = 45%). This finding maybe a consequence of the angiogenetic activity of BMP-2 that has been proved previously by various authors [20,21,22], and may be related to a more rapid formation of blood vessels during the inflammation response when BMP-2 is present, and thus lead to the observed high irregularity of the extent of the inflammatory response. However, as a whole, the total inflammatory response remains the same in all experimental groups (Figure 3).

In the literature, it is described that in the subcutaneous tissue of rats, close to the skin, this area is biomechanically very instable, due to continuous skin muscle activities which are associated with irregular mechanical forces to occur, whereas in deeper areas near the lumber spine muscles, less biomechanical instability is present in the associated tissues [17]; thus, the implanted materials are physiologically exposed at the skin side and at the lumbar body side to differential mechanical force fields with differential instability conditions. However, no major difference were observed respecting the extent of inflammation around the implanted materials at the different site, minor differences respecting thickness of local inflammation and its variance was found to be different. The most surprising finding in this study is the fact that the presence or absence of BMP-2 has no effect on the extent of the initial acute inflammatory response.

From studies in various animal models, BMPs are known to have species-specific osteoinductive dose requirements [23]. For example, in 2002, ACS/rhBMP-2 was FDA-approved as an autograft replacement for interbody spinal fusion procedures in human patients (at a concentration of 1.5 mg/cc) [24]. The BMP-2 concentration necessary for inducing consistent bone formation is substantially higher in nonhuman primates (0.75–2.0 mg/mL) than in rodents (0.02–0.4 mg/mL) [23]. In a recent publication, Luginbuehl et al. [25] found that 25 µg/mL in rodents, 50 µg/mL in dogs, 100 µg/mL in non-human primates and 800 µg/mL in humans, are quite different optimal osteoinductive BMP-2 concentrations, compared to the presently use clinical setting (0.75 and 1.5 mg/mL BMP-2) [26].

In a study of Lee et al. [10], the total amounts of BMP-2 used were 10 and 20 µg, and were diluted to 1 and 2 mg/mL, for addition to the ACS carrier, and resulted in a final BMP-2 /ACS carrier concentration of 3.3 and 6.67 mg/g for use. These authors found the inflammatory response to this construct not only to be dependent on the presence of BMP-2, but also proportionally related to its concentration. In our study, we used a total BMP-2 amount of 20 µg, dissolved and diluted to 1 mg/mL, and resulting in an ACS/BMP-2 carrier concentration of 10 mg/g, i.e., used BMP-2 in the same order of magnitude. However, we were unable to observe any additional pro-inflammatory response by the presence of BMP-2, as described by other authors [10,27]. Thus we conclude that the primary factors leading to the inflammatory response in the body are actually associated with the carrier itself and its chemical properties, but not to the presence of BMP-2. The materials used and the experimental conditions chosen in our study were the same (BMP-2, collagen) or quite similar (experimental conditions) to these previous studies [10].

It was interesting to find that in the different local areas (skin vs. lumbar body site), the thickness of the inflammatory response was indeed significantly different (Figure 4) and/or of high variability (see discussion above). We hypothesized that at sites of higher blood vessel densities on body side, we would expect more inflammation to occur, since inflammatory responses are dependent on the presence of an extensive blood vasculature, and would expect less inflammation at sites where the blood vessel density is lower. Since this was not the case in our study (see Figure 5), and this factor obviously overpowered by another biological influence, we attribute this finding to a higher biomechanical stability condition on the site with thicker inflammatory response, i.e., on the skin side. As Figure 5 illustrates in the group with dehydrated collagen sponges without BMP-2 and wet collagen sponges with BMP-2, the blood vessel density at the body side is significantly higher than that of the skin side. In the group with a dehydrated collagen sponge with BMP-2 and wet collagen sponges without BMP-2, the thickness of the inflammation zone between these two topographical sites did not show a significant difference, which would not be expected if the suggested hypothesis would be operative. The difference in inflammation thickness may thus be related to other factors, such as discussed above and in a recent review article of James et al. [5], in which the authors describe that specific anatomic locations can be associated with distinctive adverse events to implanted materials.

We thus conclude that according to our experimental findings the use of BMP-2 is not associated with the enhancement of pro-inflammatory effects in the initial phase of scaffold material implantation. The acute inflammatory response appears to be triggered predominately by the carrier material itself, its chemistry and physical properties, irrespective of the presence of BMP-2 or its absence. The aforementioned unwanted side effects, such as postoperative inflammatory swelling [8,9], possibly attribute to the carrier material itself, not by the BMP-2. As for a surgeon, should be very careful to select an optimal carrier for BMP-2. Given the fact that BMP-2 has been described by several authors to have an attenuating effect on inflammatory responses in the later phases of the implantations [14], it is actually not surprising that we are unable to confirm that BMP-2 would have a pro-inflammatory function.

## 4. Materials and Methods

### 4.1. Animal Preparation

Twelve eight-week-old male SD rats (mean weight 230 g, range from 190–250 g) were used in this study and divided into 4 experimental groups (*n* = 6 samples per group). ACSs (Medtronic Sofamor Danek, Memphis, TN, USA) were cut into identically sized circular samples (8 mm diameter). The experimental groups were defined as follows: Group1: ACS + 20 µL sterile water, group 2: ACS + 20 µL BMP-2 solution containing 20 µg BMP-2 (The dosage of BMP-2 was determined as previously described [10]); the samples of these two groups were stored under aseptic conditions overnight. Group 3: ACS + 20 µL sterile water and group 4: ACS + 20 µL BMP-2 were prepared freshly before surgery.

For induction of a general anesthesia 3% pentobarbital were intraperitoneally injected. Aseptic techniques were used during the surgical procedures. The iliac crest was used as the landmark for determining the location of the skin incision, a 25 mm posterior longitudinal incision was made bilaterally, 5–10 mm laterally from the midline. ACSs were implanted with or without BMP-2 into the subcutaneous space of the lumbar back. Right after implantation, the soft tissues were repositioned and the wound was closed using standard non-resorbable suture materials. The wound was then disinfected with 10% povidone-iodine. Animals were kept at 23 °C ambient temperature conditions until awakening. 

### 4.2. Animal Husbandry

The SD rats were kept in animal experiment center (Zhejiang Chinese Medical University Laboratory Animal Research Center, Hangzhou, China). Temperature for keeping the SD rats was 18–23 centigrade, day/night light cycle time were 14/10 (h/h), humidity 60%–80%, sterile complete feed(Anlimo, Nanjing, China) and filtered water were freely available.

### 4.3. Tissue Processing

The rats were sacrificed on postoperative day 2, at which point the collagen samples were retrieved with the adhering/surrounding tissues and chemically fixed in buffered 10% formaldehyde solution [17] for 1 day at ambient temperature, they were rinsed in tap water, dehydrated in ethanol and embedded in methylmethacrylate [14]. Using a Leica diamond saw (Leco VC-50, St. Joseph, MO, USA), the tissue blocks were cut into 5–7 slices, 600-μm-thick and 1mm apart, according to a systematic random sampling protocol [28]. All slices were then glued to plastic specimen holders and ground down to a final thickness of 80–100 μm. They were then surface-polished and surface-stained with McNeal’s Tetrachrome, basic Fuchsine and Toluidine blue, according to the publication of Schenk et al. [29].

### 4.4. Histomorphometry

#### 4.4.1. Sample Volume and Volume of Inflammation

The sections were photographed at a final magnification of ×40 in a Nikon light microscope (Eclipse 50i Microscope, Tokyo, Japan), and photographic subsampling performed according to a systematic random-sampling protocol [28]. Using the photographic prints, the volume of the implants and the inflammation areas (associated with each sample) were determined by point counting [30], respecting stereological principles. The final volumes were estimated using Cavalieri’s principle [28].

#### 4.4.2. Thickness of Inflammation Volume

It was visually observed that the inflammation thickness of the periimplant inflammation zone was different when comparing the skin side and lumber body side areas. It therefore was decided to measure the thickness of the skin side and the opposite location at the body side by drawing parallel lines across the sample and vertically to its surface; thickness measurements were performed along these lines between the implant surface boundary and the end of the inflammation zone.

#### 4.4.3. Blood Vessel Density

In dry ACS and ACS/BMP-2 group, using the photographic prints (magnification ×40), areas for high magnification imaging (×200) were chosen according to a systematic random protocol to be photographed and for morphometrical determination of the area density of blood vessels, again both on the skin side and on the opposite body side [28].

#### 4.4.4. Statistical Analysis

Independent *t*-tests were applied to the data to obtain specific comparisons between experimental and control groups of the histomorphometrical data. All statistical analyses were performed with SPSS^®^ 21.0 software (SPSS, Chicago, IL, USA), and statistical significance was defined as *p* < 0.05.

## 5. Conclusions

It is the collagen carrier itself that is the determining factor in eliciting and regulating the degree of the inflammatory response in the acute phase after implantation of an ACS/BMP-2 carrier construct in the bodily environment. This finding suggests that further development and optimization of the carrier material may be a promising way to reduce in the future the incidence and extent of the early inflammatory response as an unwanted side-effect in the soft tissue reactions around this type of implants.
